# Administration of Human Non-Diabetic Mesenchymal Stromal Cells to a Murine Model of Diabetic Fracture Repair: A Pilot Study

**DOI:** 10.3390/cells9061394

**Published:** 2020-06-03

**Authors:** Luke Watson, Xi Zhe Chen, Aideen E. Ryan, Áine Fleming, Aoife Carbin, Lisa O’Flynn, Paul G. Loftus, Emma Horan, David Connolly, Patrick McDonnell, Laoise M. McNamara, Timothy O’Brien, Cynthia M. Coleman

**Affiliations:** 1 Orbsen Therapeutics Ltd., Galway City H91 EFD0, County Galway, Ireland; luke.watson@orbsentherapeutics.com (L.W.); loflynn@avectas.com (L.O.); paul.loftus@orbsentherapeutics.com (P.G.L.); emma.horan@orbsentherapeutics.com (E.H.); timothy.obrien@nuigalway.ie (T.O.); 2 College of Medicine, Nursing and Health Sciences, School of Medicine, Regenerative Medicine Institute, National University of Ireland Galway, Galway City H91 W2TY, County Galway, Ireland; xizhe.chen@nuigalway.ie (X.Z.C.); aideen.ryan@nuigalway.ie (A.E.R.); fleminai@tcd.ie (Á.F.); acarbin9@gmail.com (A.C.); 3 CÚRAM Centre for Research in Medical Devices, College of Medicine, Nursing and Health Sciences, School of Medicine, National University of Ireland Galway, Galway City H91 W2TY, County Galway, Ireland; laoise.mcnamara@nuigalway.ie; 4 Biomedical Engineering, College of Science and Engineering, National University of Ireland Galway, Galway City H91 HX31, County Galway, Ireland; david.connolly@nuigalway.ie (D.C.); patmcd22@gmail.com (P.M.)

**Keywords:** bone, fracture repair, mesenchymal stromal cell, diabetes, mesenchymal stem cell

## Abstract

Individuals living with type 1 diabetes mellitus may experience an increased risk of long bone fracture. These fractures are often slow to heal, resulting in delayed reunion or non-union. It is reasonable to theorize that the underlying cause of these diabetes-associated osteopathies is faulty repair dynamics as a result of compromised bone marrow progenitor cell function. Here it was hypothesized that the administration of non-diabetic, human adult bone marrow-derived mesenchymal stromal cells (MSCs) would enhance diabetic fracture healing. Human MSCs were locally introduced to femur fractures in streptozotocin-induced diabetic mice, and the quality of de novo bone was assessed eight weeks later. Biodistribution analysis demonstrated that the cells remained in situ for three days following administration. Bone bridging was evident in all animals. However, a large reparative callus was retained, indicating non-union. µCT analysis elucidated comparable callus dimensions, bone mineral density, bone volume/total volume, and volume of mature bone in all groups that received cells as compared to the saline-treated controls. Four-point bending evaluation of flexural strength, flexural modulus, and total energy to re-fracture did not indicate a statistically significant change as a result of cellular administration. An ex vivo lymphocytic proliferation recall assay indicated that the xenogeneic administration of human cells did not result in an immune response by the murine recipient. Due to this dataset, the administration of non-diabetic bone marrow-derived MSCs did not support fracture healing in this pilot study.

## 1. Introduction 

Type 1 diabetes mellitus (T1DM) is an autoimmune disorder in which the immune system destroys insulin-producing beta cells, leading to elevated blood glucose levels. Over time, poorly controlled diabetes can lead to glucose toxicity, inflammation, and a variety of serious complications, including cardiomyopathy, ulcerating wounds, retinopathy, critical limb ischemia, nephropathy, neuropathy, and osteopathy. Patients with T1DM can develop early onset osteopenia or osteoporosis [1,2] and, as a consequence, have an increased risk of fracture [3,4].

Long bone fractures in diabetic patients take up to 163% longer to heal than non-diabetic fractures [5], resulting in an increased risk of complications including delayed reunion, non-union, or pseudoarthrosis. Analogous bone abnormalities are observed in pre-clinical models of T1DM that also exhibit bone loss [6,7]. Accordingly, the bones of these animals have decreased mechanical integrity, decreased mineral content [8], and inferior fracture healing potential [9]. The diabetic fracture callus contains reduced expression of chondrogenic markers and a decreased callus cross sectional area [10], indicative of reduced immunoregulatory and differentiation potential of reparative bone marrow progenitor cells. Compounding this imbalance, fractured streptozotocin (STZ)-treated mice have a 78% increase in osteoclast number, resulting in an early turnover of callus cartilage and suppression of the deposition of quality repair bone [11].

It is reasonable to hypothesize that the root cause of faulty diabetic bone homeostasis and fracture repair is a reduced population of bone marrow progenitor cells and/or their dysfunction. Mesenchymal stromal cells (MSCs) isolated from pre-clinical models of T1DM are documented to have reduced viability, suppressed proliferative rates, and inhibited osteogenic potential [12]. As reviewed by Mahmoud et al. [13], little is known about bone marrow-derived MSCs isolated from humans living with T1DM except that they have a comparable colony forming potential and growth kinetics to MSCs isolated from non-diabetic individuals [14].

Currently, there is no specific treatment for individuals living with diabetes with impaired bone healing. Equivalent slow-healing or non-union fractures in healthy subjects are treated by surgical fixation and autologous bone/marrow grafting. However, with the inflammation and reduced healing capacity associated with diabetes, these interventions are unattractive for the individuals living with diabetes. Here it was hypothesized that the administration of non-diabetic, adult human bone marrow-derived MSCs would enhance fracture repair. Human bone marrow-derived MSCs were therefore locally introduced to femoral fractures in STZ-induced diabetic C57/B6 mice, and the mineral composition and mechanical behavior were assessed as a measure of the quality of de novo bone eight weeks later. 

## 2. Materials and Methods

### 2.1. Mesenchymal Stromal Cell Isolation and Culture Expansion

Bone marrow was collected from three consenting adult volunteers aged 25.6 ± 1.1 years (mean ± SEM) according to the Galway University Hospitals ethical approval C.A. 02/08. Bone marrow was drawn from the iliac crest into heparinized collection tubes. The marrow was then diluted in phosphate buffered saline (PBS) and layered over Ficoll-Paque PLUS for isolation of the mononuclear cells by density gradient centrifugation. Adult human bone marrow-derived MSCs were cultured in α-minimal essential medium (α-MEM), with 10% fetal bovine serum (FBS), 100 U/mL penicillin, 100 μg/mL streptomycin, 1% non-essential amino acids (NEAA), and 1 ng/mL fibroblast growth factor (FGF-2) in an incubator at 37 °C with 2% O_2_ and 5% CO_2_ at 90% humidity. 

A subset of P_0_ cells was used for colony-forming unit–fibroblast (CFU-F) assays. The colonies were preserved using 95% methanol, washed twice in PBS, and stained in a 0.5% crystal violet solution. The stained colonies were counted, serving as the basis for calculating MSC expansion potential. Cell doubling rates were calculated based on a logarithmic scale of the fold increase between cells plated and cells harvested. 

### 2.2. Mesenchymal Stromal Cell Phenotypic Characterization

The following monoclonal antibodies were used for the characterization of MSCs: CD45-FITC (BD Biosciences clone HI30, dilution 1:100), CD34-PE (BD Biosciences clone 581, dilution 1:5), CD73-PE (BD Biosciences clone AD2, dilution 1:20), CD90-PE (BD Biosciences clone 5E10, dilution 1:100), MHCI-PECy7 (BD Biosciences clone G46-2.6, dilution 1:20), CD80-PECy7 (BD Biosciences clone L307.4, dilution 1:20), CD105-PE (Serotec clone SN6, dilution 1:10), MCHII-FITC (Biolegend clone L243 at 1:200), and CD86-AF488 (Biolegend clone IT2.2, dilution 1:20). For staining, the cells were washed with FACS buffer (PBS containing 1% FBS and 0.1 % NaN_3_). The antibodies were diluted in 50 μL of FACS buffer, combined with 1 × 10^5^ cells/sample and incubated for 30 min at 4 °C. The unbound antibody was removed by washing twice with FACS buffer. The cells were resuspended in FACS buffer for analysis on a BD FACS Canto. Compensation parameters were established on the FACS Canto using appropriately single stained cells or compensation beads and fluorescence minus one (FMO) controls. Propidium iodide (PI) was used as a viability dye to exclude dead cells and debris from analysis. The data were analyzed using the FlowJo software version 10 (Tree Star Inc, OR, USA) .

### 2.3. Mesenchymal Stromal Cell Differentiation

Adipogenic differentiation: MSCs were plated into a 6 well plate at 200,000 cells per well and incubated at 37 °C with 5% CO_2_ at 90% humidity. After 24 h, the conditioned media was removed from the treated wells and 3 mL of induction media (10% FBS, 10 μg/mL insulin, 100 U/mL penicillin, 100 μg/mL streptomycin, 1 μM dexamethasone, 200 μM indomethacin, 500 μM 3-isobutyl-1-methyl-xanthine in high glucose DMEM) was added. The media on control wells was replaced every 3 days with fresh expansion media. The media on treated wells was changed to maintenance medium (10% FBS, 10 μg/mL insulin, 100 U/mL penicillin, 100 μg/mL streptomycin, and high glucose DMEM) for 24 h then back to induction media for 3 days. The cycle of 1 day of maintenance media and 3 further days of induction media was continued for 8 days, followed by maintenance media for 5–7 days.

At completion, the cultures were washed and fixed in 10% neutral buffered formalin. A 0.18% Oil Red O working solution was prepared with 6 parts stock Oil Red O (0.3 g Oil Red O in 100 mL 99% isopropanol) in 4 parts distilled water, then placed on the cells for 5 min. Excess stain was removed with 60% isopropanol. Bound Oil Red O stain was extracted with 100% isopropanol and its absorbance detected on a Wallac 1420 Victor3 at 490 nm.

Osteogenic differentiation: MSCs were cultured in a 12 well plate at 100,000 cells per well and incubated at 37 °C with 5% CO_2_ at 90% humidity. After 18 hours, osteogenic media (10% FBS, 10 mM β-glycerophosphate, 100 U/mL penicillin, 100 μg/mL streptomycin, 50 μM ascorbic acid 2-P, 100 nM dexamethasone, and low glucose DMEM) was added to the treated wells. Undifferentiated controls were maintained in MSC expansion medium. The media was changed every 3 days for 10–14 days. 

To quantify calcium deposition, the cultures were scraped from the plate into 1 mL of 0.5 M HCl and the calcium extracted overnight at 4 °C. The dissolved calcium was quantified with a Stanbio Calcium assay kit as per the manufacturer’s instructions.

Chondrogenic differentiation: MSC cultures were trypsinized and resuspended in complete chondrogenic media (CCM; comprised of 50 μg/mL ascorbic acid 2-phosphate, 40 μg/mL L-proline, 6.25 μg/mL bovine insulin, 6.25 μg/mL transferrin, 6.25 μg/mL selenous acid, 5.33 μg/mL linoleic acid, 1.25 μg/mL bovine serum albumin, 1 mM sodium pyruvate, 100 U/mL penicillin, 100 μg/mL streptomycin, 1% NEAA, 100 nM dexamethasone in high glucose DMEM with TGF-β3 at 10 ng/mL). Cells were distributed into a 96 well plate at 250,000 cells per well and centrifuged at 400 G for 5 min to pellet the cells, followed by incubation at 37 °C with 5% CO_2_ at 90% humidity. The media was replaced every 3 days for 21 days. On the day of harvest, the media was removed and pellets were washed with PBS and stored dry at −20 °C.

To quantify the GAG content, pellets were digested in DMMB dilution buffer (50 mM sodium phosphate, 2 mM N-acetyl cysteine, 2 mM EDTA, and water, pH 6.5) supplemented with 1 mg/mL papain. Two hundred microliters of digestion solution were added to each pellet and incubated overnight at 60 °C, followed by vortexing. Chondroitin-6-sulphate (C6S) standards were created with 0.4 mg/mL of C6S in dilution. Two hundred microliters of DMMB stock solution was added to each standard or sample in the plate and incubated at room temperature for 5 min. The absorbance of each sample was detected at 595 nm.

To quantify the DNA content in each pellet, PicoGreen solution was diluted 200-fold in TE (10 mM Tris-HCl, 1 mM EDTA, pH 7.5). DNA standard stock solution was diluted 50-fold to give 2 μg/mL. Samples were diluted 1:100 with TE and 100 μL of standard or the sample was loaded into a black 96 well plate. One hundred microliters of PicoGreen working solution was added to each well and the plate was incubated at room temperature for 2–3 min. The fluorescence was detected at excitation and emission wavelengths of 485 nm and 535 nm.

### 2.4. Pre-Clinical Model of Diabetic Fracture Repair

All pre-clinical experiments were reviewed and approved by the university animal welfare and ethics committees as well as the Irish Health Products Regulatory Authority (AE19125/P004). Diabetes was induced in male C57/B6 mice at 8–10 weeks of age with five daily intraperitoneal injections of 50 mg/kg streptozotocin in 0.1 M citrate buffer [15]. Blood glucose content and weight were monitored weekly by tail vein blood sampling. A mouse was deemed diabetic after three consecutive weeks with a blood sugar content of over 13 mmol/L. Only diabetic mice were enrolled into the study. 

Analgesics, such as carprofen, lidocaine, adrenaline, and buprenorphine, were administered as appropriate. The mouse was placed in a supine position with a continual flow of 2.5% isoflurane mixed with oxygen at 5 mL/min to maintain anesthesia. All hair was removed from the limb and the knee was flexed to 90° and secured in place. Routine disinfection and draping ensured sterility. A 6 mm straight incision was created on the medial knee. Under magnification, a blunt separation was made along the medial patella ligament to expose the joint capsule. By entering the anterior joint capsule, the patella was exposed and dislocated laterally. With an electrical drill, a pilot hole was created between the femoral condyles (0.3 mm × 1 mm) in line with the marrow cavity of the femur. A 27-gauge needle was inserted through the pilot hole into the femoral cavity until encountering resistance. The external portion of the needle was removed after entering 1.5–1.8 cm into the marrow cavity, such that the pin lay flush with the femoral groove. The patella was returned to position and the articular capsule was closed with two biodegradable 6-0 Vicryl sutures.

The anesthetized mouse was then positioned into a custom made fracture device as described by Marturano et al. [16]. Only transverse, complete fractures without any associated bone chips were enrolled into the study. Daily healthy checks were conducted for 5–7 days after surgery to identify and intervene upon observing any sign of distress, infection, or immobility.

Two days after fracture creation, 250,000 or 500,000 MSCs in 50 µL of saline were directly administered to the fracture site with radiographic guidance. The cells had completed 2 passages before in vivo administration. The negative control group received a saline vehicle. The cell doses utilized in this study were based upon the positive results published by Prof. HJ Anders as part of the REDDSTAR collaboration. Fracture healing was monitored weekly with radiography. Fifty-six days after cell administration the mouse was sacrificed by carbon dioxide inhalation. Both the fractured and contralateral femurs were harvested, wrapped in saline soaked gauze, and frozen at −20 °C for micro-computerized tomography (µCT) followed by mechanical testing. 

### 2.5. In Vivo Assessment of Cellular Biodistribution

As described above, the mouse was rendered diabetic with STZ injections and a stabilized fracture was created. Two days post-fracture, a single dose of 250,000 adult human bone marrow-derived MSCs in 50 µL saline was locally delivered into the fracture. The negative control group received 50 µL of saline delivered directly to the fracture. Whole mouse organs were harvested immediately following cell administration (*n* = 4 cell treated, *n* = 4 saline treated) or Day 1 (*n* = 4 cell treated), Day 2 (*n* = 5 cell treated), Day 3 (*n* = 5 cell treated), and Day 7 (*n* = 5 cell treated) post-MSC administration. 

Genomic DNA (gDNA) isolation, purification and qPCR analysis of human DNA (hDNA) Alu sequences, and calculation of retained human cellular quantities were conducted as previously described [17].

### 2.6. Micro-Computed Tomography

Along the short axis of the diaphysis, the central point of the fracture was identified, as well as scanning 150–250 sections above and below with 55 kVp, a current of 200 µA, and a 500 ms integration time, producing a resolution of 10 µm^3^ voxel size. Scans ranged from 300 to 500 slices, encompassing the full fracture callus. The image was analyzed using Scanco Medical software to quantify mineral content, bone volume, bone mineral density, total volume, and bone surface area. The sample was contoured to define the tissue boundaries, the background noise reduced with a Gaussian filter (sigma 0.8, support 1.0), and a fixed, global threshold of 220 utilized to create histograms in all samples. The first, middle, and last slice was exported and the major and minor diameter measured with the Image J software (National Institutes of Health, Bethesda, MD, USA). Calculating the volume of mature bone in the callus was achieved by determining the volume of each sample with a density greater than 1000 mgHA/m^3^.

Bone tissue was segmented from non-bone tissue using the thresholding algorithm provided by the µCT manufacturer, and the output density data (Hounsfield Units) were converted to mineral content g/cm^3^. Mineral content measures were determined from specific regions (*n* = 4 per animal/per group) that were selected for analysis and conformed to a volume of interest.

### 2.7. Mechanical Testing

Femurs were thawed while on ice before loading into a custom made four-point bending apparatus as previously described by Coleman et al. [18] and flexed to failure using a 100 N load cell. The supports of the flexural fixture spanned the length of the femur (L_tot_ = 13 mm). The loading platens were positioned centrally relative to the supports such that the distance from each support to the nearest loading platen was L_1_ = 5 mm. A constant rate of axial displacement was applied to the loading platen perpendicular to the long axis of the bone at 0.166 mm per second. The second moment of area (I) was calculated from the outer major (B) and minor (D) diameter and the inner major (b) and minor (d) diameter of the femur using the equation below [19].

(1) $$I=\frac{\pi \left(BD^{3}-bd^{3}\right)}{64}.$$

The modulus (E) was calculated from the slope of the force–displacement curve such that incorporating the change in force at the points at which the modulus was calculated (F), the distance between the support and loading platen (L_1_), deflection (δ), and the second moment of area (I).

(2) $$E=\frac{FL_{1}^{3}}{48I\delta }.$$

The ultimate strength (S) was calculated from the peak force (F_ult_) and half the minor outer diameter of the callus (C) as follows:

(3) $$S=\frac{F_{ult}L_{1}C}{4I}.$$

The total energy required to re-fracture the femur was calculated by integrating travel (δ = x_2_ − x_1_) by force (F = y_1_ + y_2_) such that

(4) $$mJ=\frac{\delta F}{2}.$$

The resultant data was presented demonstrating individual biologic replicates as well as the mean ± the standard error of the mean. 

### 2.8. Xenogeneic Re-stimulation Assays 

Using the same animals described in Section 2.4, the spleen and draining lymph nodes from animals receiving saline (*n* = 3) or the injection of 500,000 MSCs (*n* = 3) were isolated at sacrifice, as previously described [20]. Lymphocytes isolated from 3 animals per treatment group were investigated using technical duplicates. Moreover, 1 × 10^5^ CFSE-labeled lymphocytes from each animal (responder cells) were added to a well of a 96-well plate. Un-irradiated human MSCs were co-cultured with the lymphocytes as stimulator cells. The co-cultures were incubated for 5 days at a ratio of 1:20 and 1:5; stimulator (MSC): responder (lymphocytes). After 5 days at 37 °C in a humidified incubator, the proliferation and activation of responder lymphocytes were determined by flow cytometry using a BD FACs Canto A. The percent proliferation was calculated and comparisons were made between lymphocytes isolated from saline-treated animals and cell-treated animals, then statistically compared using a non-parametric Mann–Whitney U-test and Kruskal Wallis one-way analysis of variance. Lymphocytes from MSC treated or saline-treated animals cultured without MSCs served as a negative control while PHA-stimulated lymphocytes served as a positive control. 

### 2.9. Statistical Analysis

Statistical analysis of outcome data was performed with GraphPad Prism version 5.03 (San Diego, CA, USA). Power calculations were performed with MiniTab 17 (Coventry, UK).

## 3. Results

### 3.1. Bone Marrow-Derived MSC Characterization

**Figure 1:**
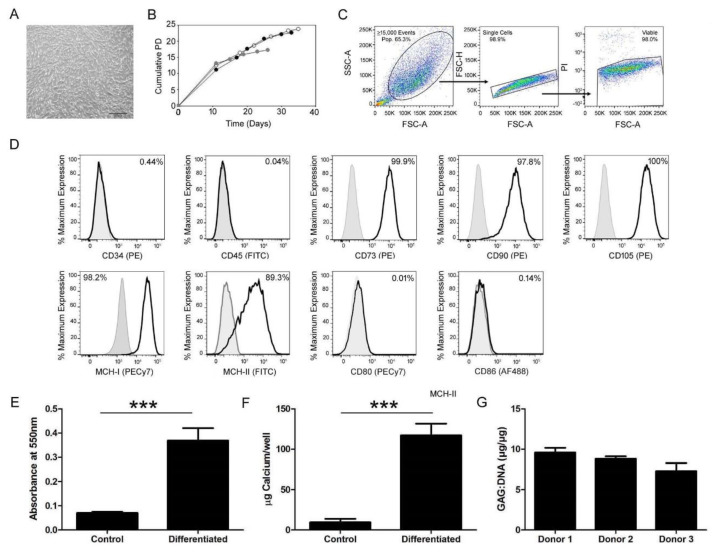
Characterization of bone marrow cell isolates demonstrated a mesenchymal phenotype with tri-lineage potential. To validate the isolation of mesenchymal stromal cells (MSCs) from the bone marrow, morphologic and phenotypic characterizations were conducted. (**A**) The isolated MSCs maintained an elongated, fibroblastic appearance in vitro culture. The scale bar represents 500 µm. (**B**) With culture expansion, the cumulative population doublings (PD) were calculated and monitored to ensure continual exponential proliferation at the time of in vivo transplantation ensuring treatment of the fracture with an active population of cells. The expansion curves of three independent donors are displayed in this graph, represented by solid black, gray, or open white circles. (**C**) After gating flow cytometric data to include only viable single cells, (**D**) representative samples, low expression of CD34 (0.44%) and CD45 (0.04%) and high expression of CD73 (99.9%), CD90 (97.8%), and CD105 (100%), indicated the isolation of a population of cells conforming to the ISCT standards. The cells were also positive for MHC-I (98.2%) and MHC-II (89.3%), but negative for co-stimulatory CD80 (0.01%) and CD86 (0.14%). Gray lines indicate the isotype control while black lines indicate an antibody stained sample. Tri-lineage differentiation in (**E**) adipogenic, (**F**) osteogenic, and (**G**) chondrogenic conditions and subsequent biochemical analysis revealed an increase in (**E**) Oil Red O retention in cytoplasmic lipid droplets, (**F**) calcium, and (**G**) GAG incorporation into the extracellular matrix over undifferentiated control cultures, indicating the multi-lineage potential of the isolated cell population. A Welch’s unequal variances T-Test was used to assess differentiation (**E**, **F**).

To ensure the primary cultures isolated from adult human bone marrow were a population of MSCs, their morphology, expansion characteristics, cell surface phenotype, and tri-lineage potential were confirmed in vitro. Upon culture, the isolated cells retained the elongated, fibroblastic morphology characteristic of MSCs (Figure 1A). They proliferated in a manner characteristic of MSCs with an exponential phase of expansion through early passages, slowing with time to a plateau at later passages (Figure 1B). Although there were differences between donors, all cultures were proliferative when they were prepared for administration to the fracture (following P_2_). Flow cytometric analysis (Figure 1C) of the primary culture for the expression of International Society of Cell & Gene Therapy (ISCT)-standardized cell surface markers [21] indicated the low expression of CD34 and CD45 and the high expression of CD73, CD90, and CD105 (Figure 1D). The MSCs were also found to be positive for both MHC-I and MHC-II, but negative for the co-stimulatory molecules CD80 and CD86 (Figure 1D). 

In vitro culture of the primary cell isolates in adipogenic, osteogenic, and chondrogenic culture confirmed the tri-lineage potential of the primary bone marrow isolates. Biochemical analysis of Oil Red O dye retention in the cytoplasmic lipid vesicles (Figure 1E) and calcium retention in the extracellular matrix (Figure 1F) was statistically enhanced (*p* < 0.0001) over undifferentiated controls, demonstrating cellular differentiation into adipocytes and osteocytes, respectively. When quantifying retained Oil Red O, extracts from adipogenically differentiated cultures exhibited a mean absorbance of 0.369 nm while undifferentiated cultures retained very little stain at 0.070 nm. Osteogenically differentiated samples incorporated a mean of 117.022 µg of calcium/well while undifferentiated controls retained 12.14 µg of calcium/well. Chondrogenic differentiation in high density pellets (Figure 1G) resulted in the secretion and retention of GAGs in the extracellular matrix of three biologic replicates. The repeated observation of over 5 µg of GAG per µg of DNA within the pellet culture positively indicated the differentiation of the isolated cell population into mature chondrocytes. 

### 3.2. Murine Model of Diabetic Fracture Repair

**Figure 2:**
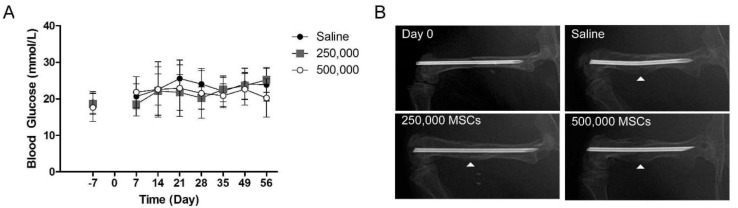
Diabetic mice retained a fracture callus 8 weeks after cellular administration. Diabetes was induced in 8+-week-old male mice with repeated daily administration of STZ, followed by 3 weeks of maintenance upon the onset of diabetes. (**A**) Weekly tail vein blood glucose monitoring demonstrated the onset of diabetes before fracture creation (−7 days) followed by sustained, elevated blood glucose for the duration of the study. Fracture induction (0 days) or the administration of MSCs (2 days) did not result in a change of blood glucose levels. (**B**) Radiographs were obtained throughout the study. On the day of induction (Day 0), a fixated, transverse fracture was required for entry into the study. After 56 days, radiographic analysis of diabetic fractures treated with saline, 250,000 MSCs, or 500,000 MSCs revealed preliminary bone bridging with the persistent presence of a reparative callus (indicated by the white arrow head).

Diabetes was induced in male C57/B6 mice with repeated, daily injections of STZ. Indicative of the onset and severity of diabetes, the glucose content of tail vein blood samples was monitored for three weeks before fracture induction and throughout the eight weeks of fracture repair. Blood glucose levels over 13 mmol/L were considered hyperglycemic. All mice enrolled into the study were hyperglycemic for three weeks before fracture induction (0 days), on the day of cellular administration at two days (Figure 2A) to the completion of the investigation. The elevated blood glucose was comparable in all treatment groups throughout the duration of the study.

Throughout the in vivo phase of the study, radiographs were obtained to visualize the progression of fracture repair. A notable callus was retained in all mice in all treatment groups 56 days following fracture (Figure 2B). Preliminary bone bridging was evident in all animals. However, a large circumferential reparative callus containing sites of mineralization was consistently retained, indicating non-union. 

### 3.3. Cellular Retention and Distribution

To quantify the retention and distribution of MSCs following local administration to the injury site, a fracture was created in diabetic mice and 250,000 MSCs were locally delivered. Femoral fractures treated with the saline vehicle alone served as a control. In the negative control group (day 0 saline treated), no human DNA (hDNA) was present in any assayed organs within the detection limits of this assay demonstrating the absence of hDNA contamination external to that present in the therapeutic MSCs (Table 1). 

hDNA was detected in murine organs on the day of cellular administration and for three days following (Table 1). At the Day 0 and Day 1 time points, it was determined that MSC retention was confined locally to the bone and adjacent muscle. Small intestines, spleens, livers, stomachs, pancreases, kidneys, and lungs were consistently negative for the presence of hDNA on both days. However, some hDNA was detectable in the large intestine and heart in one of four biologic replicates on Day 1. 

Two and three days following MSC delivery, the greatest quantity of hDNA was noted within the muscle adjacent to the fracture site, reducing in quantity at the later time point. hDNA was identified within the kidney of one animal on Day 2 following cellular administration. hDNA was also detected in the bone of one animal on Day 2 and two animals on Day 3 following MSC administration. hDNA was not detected within the small intestines, spleens, large intestines, livers, stomach, pancreas, lungs, and hearts at either time point. 

No hDNA was detected within any of the organs investigated seven days after local cellular administration (Table 1).

### 3.4. MSC Administration did not Augment the Quality of De Novo Reparative Bone

**Figure 3:**
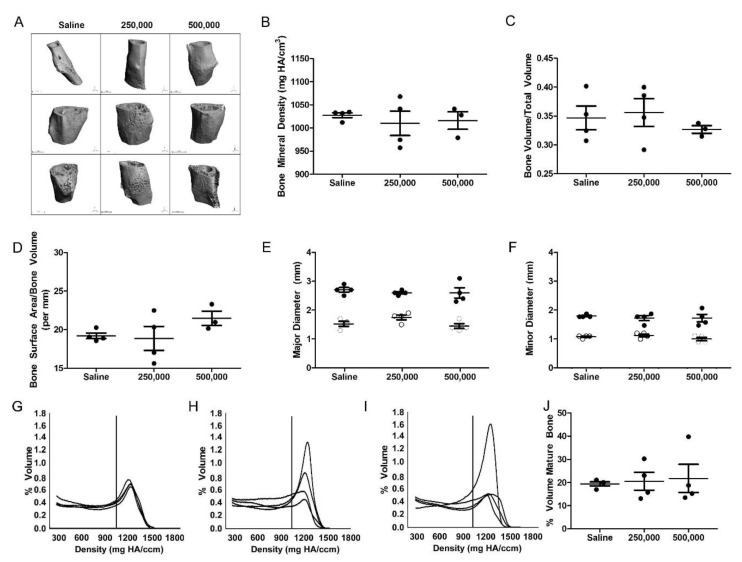
Administration of MSCs did not enhance the quality of de novo bone production. (**A**) µCT analysis of the fracture callus 56 days after cellular administration confirms the continued presence of a callus at the fracture site. The (**B**) bone mineral density, (**C**) bone volume, (**D**) bone surface area, and callus (**E**) minor and (**F**) major axis diameters remained consistent in all treatment groups. In (**E**–**F**), the solid circles indicate the outer diameter of the callus, while the white circles indicate the inner diameter of the femur. In cell-treated femurs (250,000 cells in (**H**) and 500,000 cells in (**I**)), there was an increase in mineralized mature bone content in a minority of femurs that was absent in the (**G**) saline-treated femurs, but the (**J**) mean value remained consistent across all groups. In B-D and J, the solid dot indicates *n* = 1 biologic replicate. One-way ANOVA with Bonferroni’s multiple comparison test (**B**–**F**) or Tukey’s multiple comparison test (**J**) was used to assess the resultant data.

µCT analysis of ex vivo femurs (Figure 3A) confirmed the radiographic observation (Figure 2B) of a persistent callus eight weeks after cellular administration. The callus was observed to be heterogeneous in nature, with a mineralized exterior and a non-mineralized interior. 

The bone mineral density of the repairing fracture was not significantly different for any of the treatment groups with an average of 1028 ± 10.5 mg HA/cm^3^ in the saline controls, 1010 ± 52.8 mg HA/cm^3^ in fractures receiving 250,000 MSCs, and 1016 ± 32.8 mg HA/cm^3^ in fractures treated with 500,000 MSCs (Figure 3B). Similarly, the bone volume, normalized to the total volume, was comparable in all treatment groups with 34 ± 0.04% in the saline control as compared to 35 ± 0.04% and 32 ± 0.01% in fractures treated with 250,000 or 500,000 MSCs, respectively (Figure 3C). Bone surface area normalized to bone volume was maintained at 19.2 ± 0.7 per mm in fractures treated with saline to 18.8 ± 3.1 per mm and 21.4 ± 1.6 per mm in those receiving 250,000 or 500,000 cells, respectively (Figure 3D). 

Physical characterization of the callus size demonstrated consistency in all treatment groups. The width of the outer major axis was consistently maintained at a mean of 2.7 ± 0.1 mm in saline controls as compared to 2.6 ± 0.1 mm after the administration of both 250,000 and 500,000 cells (Figure 3E). Upon measuring the inner major diameter via µCT, it was found that the average 1.5 ± 0.1 mm diameter in saline controls was comparable to that observed in femurs treated with 250,000 cells (1.6 ± 0.1 mm) or 500,000 cells (1.4 ± 0.1 mm). Similarly, the 1.8 mm ± 0.05 diameter of outer callus minor axis upon the administration of saline was comparable to that of 1.8 ± 0.1 mm following treatment with either 250,000 MSCs or 500,000 MSCs (Figure 3F). The inner minor diameter of the reparative callus was analogous in all groups at 1.1 ± 0.05 mm in saline controls, 1.2 ± 0.2 mm in fractures treated with 250,000 cells, or 1.0 ± 0.1 mm in fractures treated with 500,000 cells. 

By profiling the callus composition using µCT histograms, the mineral content was quantified. Control fractures treated with saline were shown to have approximately 19.4% ± 1.7% mature bone (>1000 mgHA/cm^3^) within the callus volume (Figure 3G,J). Fractures treated with 250,000 MSCs contained comparable quantities of mature bone in the callus (20.5% ± 7.7%) (Figure 3H,J). In a pattern similar to saline controls, diabetic fractures treated with 500,000 MSCs consistently retained only a small percentage of mature bone (21.8 ± 12.1%), as displayed in Figure 3I,J. There was no significant difference in tissue density as a result of MSC administration as compared to the control group. 

### 3.5. Mechanical Assessment of the MSC-Treated Fracture

**Figure 4:**
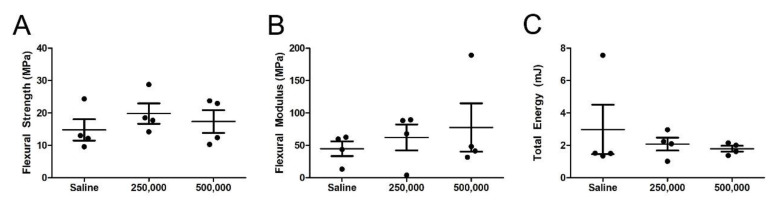
Mechanical testing did not reveal a statistically significant enhancement in integrity of the repairing fracture. Four-point bending analysis of ex vivo fractures harvested 8 weeks after cellular administration revealed comparable (**A**) flexural strength, (**B**) flexural modulus, and (**C**) total energy to elicit failure, indicating no impact as a result of cellular administration. The solid dot indicates *n* = 1 biologic replicate. One-way ANOVA with Bonferroni’s multiple comparison test was used to assess the resultant data (**A**–**C**).

Four-point bending assessment of the repairing fracture was conducted to evaluate the de novo bone’s capacity to withstand stress and bending before re-fracturing. From a load–displacement curve, the flexural strength (Figure 4A) and flexural modulus (Figure 4B) were calculated. No statistically significant differences in flexural strength were observed in diabetic fractures treated with MSCs as compared to those treated with the saline vehicle alone. The average baseline of 14.7 ± 6.5 MPa in the saline control was comparable to that of the cohort treated with 250,000 MSCs (19.8 ± 6.2 MPa) or 500,000 MSCs (17.3 ± 6.9 MPa). When evaluating the flexural modulus (Figure 4B), it was observed that the flexural modulus of the saline cohort (44.7 ± 22.6 MPa) was less than that of femurs treated with 250,000 cells (62.3 ± 40.2 MPa), although this difference was again not statistically significant. The flexural modulus of the femurs treated with 500,000 cells (40.3 ± 8.4MPa) was skewed upward due to an outlying point (at 189 MPa) identified via the Grubb’s test, but the average of clustering samples is statistically indistinct to that of the saline-treated controls.

Upon calculating the area under the load-displacement curve, it was observed that all groups required comparable amounts of energy to re-fracture the de novo bone. While a saline-treated fracture required 2.9 ± 3 mJ for callus failure, fractures treated with 250,000 or 500,000 MSCs resisted 2.0 ± 0.8 mJ or 1.7 ± 0.3 mJ of pressure before failure, respectively (Figure 4C). It was noted that the total energy calculated in the saline cohort is overestimated due to a statistical outlier identified via the Grubb’s test (7.5 mJ). 

### 3.6. Xenogeneic MSCs do not Stimulate Lymphocytic Proliferation

**Figure 5:**
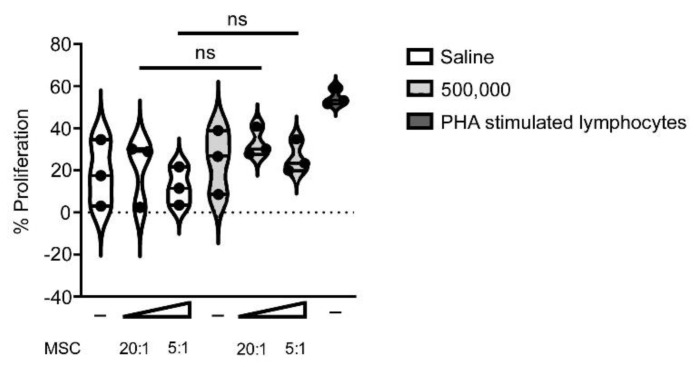
Lymphocytic re-stimulation analysis indicated no increase in pre-exposed murine lymphocytic proliferation as a result of exposure to human MSCs in vivo. Murine lymphocytes were isolated from animals treated with saline (white) or 500,000 MSC (light gray) injected into the fracture, then cultured ex vivo without MSC stimulation (-) or with MSC stimulation at a ratio of 20:1 or 5:1. PHA stimulated lymphocytes served as a positive control (dark gray). No statistically significant (ns) increase in the proliferation of lymphocytes was observed following their re-exposure to MSCs ex vivo. The resultant data was statistically compared using a non-parametric Mann–Whitney T-Test and Kruskal Wallis one-way ANOVA.

To understand if the administration of xenogeneic MSCs resulted in a murine immune response, an ex vivo re-stimulation assay was conducted (Figure 5). The baseline, non-stimulated proliferation of lymphocytes isolated from saline-treated animals and those treated with 500,000 MSCs were comparable. Co-culturing lymphocytes from the saline-treated animals with stimulator MSCs at a 20:1 or 5:1 ratio resulted in levels of lymphocytic proliferation comparable to that of the unstimulated control. Co-culturing lymphocytes from animals that received 500,000 MSCs to the fracture with stimulatory MSCs in vitro resulted in an increase in lymphocytic proliferation. The proliferation was comparable when stimulated with low (20:1) or high (5:1) concentrations of stimulatory MSCs. Due to the difference between the non-MSC treated negative controls and the PHA-stimulated positive controls, this re-stimulation assay is validated to detect a change in lymphocytic proliferation as a result of MSC-stimulation. Using a one-way ANOVA and a non-parametric T-Test, there was no statistically significant increase in lymphocytic proliferation as a result of MSC re-exposure. 

## 4. Discussion

Individuals with T1DM have an increased risk of fracture [3,4] and delayed fracture repair [5], resulting in a higher risk of delayed reunion or non-union. As the presence of T1DM results in reduced osteogenic potential in rodent progenitor cells [22,23] and in vitro models of high-glucose exposure indicate similar results with human MSCs [24], it was hypothesized that deficiencies in host progenitor cell capacity results in inhibited fracture repair. If this hypothesis is correct, the administration of healthy, allogeneic adult bone marrow-derived MSCs will enhance diabetic fracture healing. The aim of this endeavor was to test that hypothesis and to optimize MSC dosing, resulting in the greatest fracture repair efficacy. 

Refuting the hypothesis, the findings of this study demonstrated that the administration of allogeneic, non-diabetic MSCs two days following injury did not statistically enhance diabetic femoral fracture healing. Although the cells remained in situ for several days following local administration, there was no statistical evidence of greater amounts of mature bone eight weeks later. Four-point bending of the same samples did not elucidate a significant increase in flexural strength and modulus as a result of cellular administration. Together these data demonstrate that the administration of 250,000 or 500,000 xenogeneic non-diabetic cells to a murine diabetic fracture two days following injury does not significantly enhance the development of de novo bone.

Human MSCs have been investigated in pre-clinical models as an interventional therapeutic for diabetes-induced nephropathy [25,26] wound ulceration [27], retinopathy [28,29], and islet destruction [24]. As a therapy for osteopathy, the administration of rodent MSCs to healing diabetic fractures, in combination with a biomimetic scaffold or allograft, resulted in enhanced bone formation over the administration of a scaffold alone in a rodent model [30]. The xenogeneic administration of human MSCs to a leporine model of diabetes, in combination with a scaffold, indicated the promise of human MSCs to support diabetic fracture repair without an observed local or systemic inflammatory response [31]. To advance the state of the art, the objective of this investigation was to utilize the intended translational product (e.g., human MSCs), delivered simply (e.g., directly, without a scaffold or delivery device) in a proof of principal investigation to establish the potential of human MSCs to support the repair of a diabetic fracture. 

When evaluating an advanced progenitor cell therapeutic intervention in vivo, such as healthy MSC transplantation, establishing the quality of the donor MSCs is a requirement for investigational success. In this study, the MSCs isolated from healthy, non-diabetic human donors retained the anticipated fibroblastic morphology upon culture, were proliferative at the time of transplantation, and displayed all of the cell surface markers typical of a progenitor cell [21]. The cultures were osteogenic, adipogenic, and chondrogenic, establishing their tri-lineage potential.

To establish a murine model of T1DM with a pathogenesis similar to that observed in humans, STZ was delivered to mice in multiple low doses, thereby inducing pancreatic dysfunction via immune destruction [32]. In concert with hyperglycemia, the administration of STZ established diabetes-induced osteopathy before fracture induction, as evidenced by a decrease in bone volume fraction [15]. The administration of STZ resulted in an increase in blood glucose levels averaging consistently over 20 mmol/L, comparable to levels of glycaemia reported by others [15]. Interestingly, although Dong et al., Xie et al., and Sun et al. have previously reported the restoration of insulin secretion in vitro [33] and normoglycaemia upon the administration of allogeneic or xenogeneic MSCs [33,34,35], in this study, there was no observed change in glycaemia as a result of cellular administration. Perhaps the discrepancy is due to the study design. As an example, Dong et al. [34] administered 2–4 million cells per rat intravenously, a notably larger dose of cells via a different route than those examined in this investigation.

Profiling the biodistribution and retention of therapeutic cells is often a regulatory requirement for translation to the clinic. Although several published studies have shown that the majority of cells are rapidly cleared within hours following administration [36,37,38,39], contrasting publications describe the retention of transplanted MSCs within the repair tissue for several months [40], engrafting into the fracture callus and contributing in situ with an osteocytic phenotype [41,42]. In this study, it was observed that the MSCs remained predominantly at the injection site, decreasing with time for three days following administration. Ramot et al. published a comparable pattern of gradually decreasing transplanted human placental derived-MSCs following intramuscular injection [43], albeit over a longer period of time. Huang et al. [44] described retention of a small percentage of locally administered, luciferase expressing murine MSCs in the non-diabetic callus five weeks following their administration. Perhaps the discrepancy between this study and that by Huang is the presence of diabetes-induced pro-inflammatory cytokines in this investigation, which inhibit osteocytic differentiation [45].

Although deciphering the mechanism of action of the transplanted MSCs is beyond the remit of a pilot study, it is of interest to note several published investigations indicating their therapeutic potential through paracrine activities. Investigators of future pivotal studies may choose to investigate this hypothesized paracrine mechanism, especially given the short-term retention of MSCs in this model. MSCs can elicit effects on distal tissues via apoptosis [37], protein secretion [46,47], or exosomes [48,49,50] as demonstrated in models of calverial osteogenesis [46], or fracture repair [47,48,49,50]. Xenotransplantion studies demonstrate that the early neogenic osseous tissue created by donor progenitor cells changes with time as the host cells become the predominant contributors to the heterogeneous repair tissue [51,52]. Upon MSC transplantation, the host initiates an infiltration of endothelial cells and CD146+ pericytes, stimulating host angiogenesis [53] and indirect paracrine conditioning of the local environment [54] to support bone repair. Due to the conserved nature of this mechanism between MSCs of various tissue origins and mouse, rat, and rabbit models, it is likely that a comparable mechanism would apply in future investigations of diabetic fracture repair.

In the current study, femoral fracture creation was accomplished using the device and methodology previously described [16], resulting in the reproducible creation of transverse fractures. At sacrifice eight weeks after fracture induction, a mineralized callus was observed in 100% of enrolled mice regardless of treatment group, as determined by radiography and µCT imaging. In published findings using a comparable, age-matched healthy male non-diabetic C57/B6, the callus is reported to be resorbed and remodeled eight weeks post-fracture [55], demonstrating in this study the reproducible onset of fracture non-union as a result of diabetes.

Diabetes leads to the formation of smaller calluses with decreased quantities of cartilage and inhibited maturation of bone as a result of decreased cellularity and inhibited osteoblastic differentiation [9,56]. Decreased callus cellularity, specifically a reduction in CD271+ or Sca-1+ precursors [57,58] or limited numbers of chondrocytes [59,60], results in decreased or delayed growth factor production [61], impaired osteoblastic differentiation, and therefore inhibited endochondral ossification [59,60,62]. Contrary evidence demonstrated a two-fold increase in callus size [63] due to impaired cartilage resorption and decreased osteoclastic activity [64,65,66], inhibiting the process of endochondral ossification. The current study did not elucidate a change in dimensions of the major or minor axis of the callus as a result of human MSC administration. In further analyzing the composition of the callus, little mature bone was identified in saline-treated controls or the cohort average of cell treated groups. However, an increase in mature bone volume was observed in a subset of the cell-treated cohorts. Perhaps this indicates a biologic effect in a subset of recipients due to the cellular therapy; further investigation would be required to confirm this hypothesis.

Bone mineral content is a determinant of bone strength [67,68] and alterations in tissue mineral distribution within the fracture callus can thus be indicative of enhanced mechanical integrity. Increases in the degree of bone tissue mineralization are associated with a significant enhancement of overall bone strength [69]. A statistically significant change in BMD or BV/TV was not detected as a result of cellular administration, but it should be noted that the frequency distribution and sub-volume analysis of mineral content provides a higher resolution and improved understanding of mineral content and distribution that cannot be obtained by analyzing BMD alone, an outcome averaged over the entire volume of interest. Indeed, it has been previously shown that important effects of bone disease on mineral distribution may be undetectable by studies focusing solely on mean mineral content of bone tissue [70]. Previously published investigations administering transgenic murine MSCs have indicated that 500,000 allogeneic cells are sufficient to increase the BV/TV ratio as well as the flexural strength and flexural modulus, but not the total energy required to re-fracture the de novo bone [44]. Perhaps the added complication of assessing human MSCs in diabetic fracture repair requires an increase in cell dose to demonstrate an equivalent efficacy.

It is well established that the onset of diabetes induces anomalies in skeletal biomechanics, rendering uninjured long bones weaker, stiffer, and more brittle when compared to non-diabetic controls [61,71,72]. The reduction in maximum force and energy absorption to failure, tensile strength, and stiffness [9,22,71,73,74] results in an increased fracture risk. Pre-clinical models of repairing fractures also exhibit inferior failure torque, failure stress, structural stiffness, and material stiffness when compared to repairing fractures in non-diabetic individuals [75]. In this study, there was no statistically significant change in flexural strength, flexural modulus, or total energy required to re-fracture the femur as a result of cellular administration.

It could be hypothesized that the lack of efficacy of human MSCs to stimulate diabetic fracture repair in a murine model of diabetes is the result of a xenogeneic immune response. As per ISCT guidelines [21], the MSCs were characterized for MHC-I and MHC-II expression as well as the expression of co-stimulatory molecules CD80 and CD86 [76]. It was found that the MSCs were positive for both MHC molecules, but negative for CD80 and CD86. The expression of MHC-II has previously been described on clinical-grade human MSCs [77], but its expression did not affect their in vitro differentiation or capacity to suppress lymphocytic proliferation. In this study, the expression of MHC-II is most likely the result of culturing the cells with FGF [78]. However, as they do not co-express CD80 and CD86, they should have a reduced capacity to elicit a xenogeneic immune response. The data presented here tested that hypothesis at two ratios of MSCs:lymphocytes, reflecting doses higher than what is present within the fracture. A statistically significant induction of lymphocyte proliferation was not observed. It is worth noting, however, that the re-stimulation assay was assessed using non-irradiated MSCs. MSCs secrete high levels of immunomodulatory molecules [20] and in these assays the secretion of immunosuppressive molecules by the high number of MSCs may affect the immunogenicity. Therefore, the lack of human MSC efficacy to stimulate murine diabetic fracture repair was most likely not a result of a xenogeneic immune response. Future studies should investigate the possibility of interspecies incompatibility [79].

Results from this pilot study have shown that the local administration of 250,000 or 500,000 human MSC to a murine model of diabetic fracture healing was not efficacious in improving fracture healing. The relatively small sample size of this pilot study may not allow for a definitive conclusion concerning the effectiveness of MSC administration in this context. Perhaps a greater sample size would enable the identification of statistically significant trends demonstrating MSCs improve diabetic fracture repair. Still, the data obtained in this pilot study should inform the criteria for a successful future pivotal efficacy study, including ensuring outcome measures are sufficiently powered to detect differences between treatment groups. Here, the BV/TV ratio was used as the primary outcome to calculate the sample size and the power of this study because of its clinical relevance. With a sample size of *n* = 4 mice per group, this study was powered to detect a maximum difference of 0.15 in BV/TV between groups (β = 0.82). However, as shown in Figure 3C, there was a maximum mean difference of 0.0164 between the saline and 500,000 MSC treated group. In a similar study performed by Huang et al. [63], where 500,000 MSCs were directly administered to a murine model of fracture repair (*n* = 4), a statistically significant increase of 0.1 in BV/TV was seen in MSC-treated mice compared to the saline control. Due to the standard deviation in the current study, to detect a significance level α = 0.05, a targeted power β = 0.8, and a pooled SD = 0.056, a minimum of *n* = 8 mice per group would have been required to detect a maximum difference of 0.1 in BV/TV (actual power 0.85). 

## 5. Conclusions

Together, these data demonstrate that the administration of non-diabetic bone marrow-derived human MSCs does not support the development of greater quantities of mature bone within the murine callus, resulting in comparable de novo bone strength to that of the negative (saline-treated) controls. Perhaps the impact of cellular therapy in fracture repair could be further improved with sequential administration of small doses of MSCs or administration of MSCs at an earlier time point following fracture to enhance the impact of the therapeutic trophic or paracrine agents released by the cells. The inclusion of histomorphometric analysis and immunostaining to characterize the expression pattern of key osteogenic proteins (such as osteonectin or collagen 1A) would be beneficial in future pivotal studies.

## Figures and Tables

**Table 1:** Number of cells retained in 11 tissues on the day of cell delivery (Day 0) or 1, 2, 3, and 7 days post-administration. Any CT value below .002 ng on the standard curve is outside the limits of detection in this assay and is therefore interpreted as negative for human DNA (hDNA) (ND, not detected).

	Small Intestine	Spleen	Muscle	Large Intestine	Liver	Stomach	Pancreas	Kidney	Lung	Bone	Heart
Day 0	ND	ND	0.07	ND	ND	ND	ND	ND	ND	0.01	ND
	ND	ND	ND	ND	ND	ND	ND	ND	ND	253	ND
	ND	ND	365	ND	ND	ND	ND	ND	ND	ND	ND
	ND	ND	1258	ND	ND	ND	ND	ND	ND	ND	ND
Day 1	ND	ND	4450	ND	ND	ND	ND	ND	ND	7	ND
	ND	ND	3165	ND	ND	ND	ND	ND	ND	3	ND
	ND	ND	22,082	ND	ND	ND	ND	ND	ND	53	6
	ND	ND	68	19	ND	ND	ND	ND	ND	165	ND
Day 2	ND	ND	4512	ND	ND	ND	ND	158	ND	ND	ND
	ND	ND	5082	ND	ND	ND	ND	ND	ND	ND	ND
	ND	ND	2905	ND	ND	ND	ND	ND	ND	6	ND
	ND	ND	2595	ND	ND	ND	ND	ND	ND	ND	ND
	ND	ND	1378	ND	ND	ND	ND	ND	ND	ND	ND
Day 3	ND	ND	3510	ND	ND	ND	ND	ND	ND	ND	ND
	ND	ND	495	ND	ND	ND	ND	ND	ND	ND	ND
	ND	ND	416	ND	ND	ND	ND	ND	ND	13	ND
	ND	ND	ND	ND	ND	ND	ND	ND	ND	ND	ND
	ND	ND	149	ND	ND	ND	ND	ND	ND	190	ND
Day 7	ND	ND	ND	ND	ND	ND	ND	ND	ND	ND	ND
	ND	ND	ND	ND	ND	ND	ND	ND	ND	ND	ND
	ND	ND	ND	ND	ND	ND	ND	ND	ND	ND	ND
	ND	ND	ND	ND	ND	ND	ND	ND	ND	ND	ND
	ND	ND	ND	ND	ND	ND	ND	ND	ND	ND	ND
Negative Control	ND	ND	ND	ND	ND	ND	ND	ND	ND	ND	ND
	ND	ND	ND	ND	ND	ND	ND	ND	ND	ND	ND
	ND	ND	ND	ND	ND	ND	ND	ND	ND	ND	ND
	ND	ND	ND	ND	ND	ND	ND	ND	ND	ND	ND
